# Effects of Lifestyle-Integrated Active Pursed-Lip Diaphragmatic Breathing Training on the Prognosis of Chronic Heart Failure: A Retrospective Real-World Study

**DOI:** 10.3390/healthcare14162489

**Published:** 2026-08-11

**Authors:** Qi Zhou, Ying Zhang, Dan Ma, Fengjie Lyu, Suxin Luo, Shenglan Yang

**Affiliations:** 1 Department of Cardiovascular Medicine, Cardiovascular Research Center, The First Affiliated Hospital of Chongqing Medical University, Chongqing 400016, China; zhouqi202512@163.com (Q.Z.); 13139898278@163.com (D.M.); lvfjup@163.com (F.L.); 2 Department of Geriatrics, Chongqing General Hospital, Chongqing University, Chongqing 401120, China; 3 Continuing Education College, Chongqing University of Science and Technology, Chongqing 401120, China; 4 Department of Geriatrics, Laboratory of Research and Translation for Geriatric Diseases, The First Affiliated Hospital of Chongqing Medical University, Chongqing 400016, China

**Keywords:** chronic heart failure, respiratory training, lifestyle intervention, cardiac rehabilitation, retrospective real-world study

## Abstract

**Highlights:**

**What are the main findings?**

- High PLDB adherence yields clear gains in exercise capacity and cardiac function.
- PLDB training demonstrates an association with positive clinical outcomes.

**What are the implications of the main findings?**

- Lifestyle-integrated active PLDB allows for flexible, “snack-style” daily training.
- This device-independent, home-based approach overcomes traditional rehab barriers.

**Abstract:**

**Background**: Device-independent, lifestyle-integrated active pursed-lip diaphragmatic breathing (PLDB) may overcome respiratory rehabilitation barriers in chronic heart failure (CHF). This retrospective real-world study evaluated its prognostic association with functional capacity, quality of life (QoL), and major adverse cardiovascular events (MACE). **Methods**: A total of 58 CHF patients (NYHA II–IV) were categorized by 6-month post-discharge PLDB adherence into a Respiratory Training Group (RTG; *n* = 20) and Control Group (*n* = 38). Functional, structural, QoL metrics, and MACE outcomes were analyzed. **Results**: After 6 months, the RTG demonstrated significant intra-group improvements in 6 min walk test (6MWT) distance (302.00 [243.60, 408.15] m to 386.00 [339.50, 439.00] m, *p* = 0.006; baseline-adjusted between-group gain: +76.16 m), handgrip strength (*p* = 0.046), LVEF (*p* = 0.003), and scores on the Minnesota Living with Heart Failure Questionnaire (MLHFQ) (*p* < 0.001), Generalized Anxiety Disorder-7 (GAD-7) (*p* = 0.010), and Self-rating Somatic Symptom Scale-China (SSS-CN) (*p* = 0.004). Post-intervention, the RTG outperformed controls in handgrip strength (*p* = 0.012), MLHFQ (*p* = 0.001) and SSS-CN (*p* = 0.020). The RTG exhibited a significantly lower MACE incidence (5.0% vs. 42.1%, *p* = 0.003) and superior MACE-free survival (log-rank *p* = 0.005). Multivariable Cox regression confirmed high PLDB adherence was independently associated with a lower MACE risk (adjusted HR = 0.103, 95% CI: 0.014–0.779, *p* = 0.028). **Conclusions**: High adherence to a 6-month lifestyle-integrated PLDB program correlates with clinically meaningful improvements in exercise capacity, cardiac function, and QoL, and is independently associated with a lower risk of short-term MACE in CHF patients.

## 1. Introduction

Chronic heart failure (CHF), the terminal stage of various cardiac diseases, signifies structural and functional changes in the heart, leading to a complex array of symptoms, with fatigue and dyspnea being the most prevalent. Studies reveal that 59% of CHF patients experience fatigue, while over 69% suffer from dyspnea [1]. These symptoms not only restrict patients’ physical activity and daily functioning but also diminish their social interactions, resulting in decreased self-esteem and a significant rise in anxiety and depression. Furthermore, they serve as independent predictors of rehospitalization and mortality [2]. Pursed-lip diaphragmatic breathing (PLDB), a respiratory training method that combines pursed-lip breathing with diaphragmatic breathing, has emerged as an effective strategy to improve cardiopulmonary function, enhance exercise tolerance, and ultimately improve patients’ quality of life and prognosis [3,4]. Unlike traditional exercise rehabilitation, PLDB necessitates no equipment, is not constrained by location or time, and its movements are straightforward and easily learned, even by elderly patients, those with low literacy levels, and those with decompensated HF. Building upon this, lifestyle-integrated active PLDB Training offers a flexible approach, allowing patients to integrate respiratory training into their daily routines, fostering a sustainable lifestyle over the long term. This study aims to explore the impact of lifestyle-integrated active PLDB Training on cardiac function, exercise tolerance, quality of life, and prognosis in CHF patients, and identify a potentially more convenient and effective cardiac rehabilitation method for CHF patients.

## 2. Materials and Methods

### 2.1. Study Population

This retrospective real-world study was conducted at the First Affiliated Hospital of Chongqing Medical University. The clinical records and follow-up data of patients discharged from the Department of Cardiology between January 2023 and January 2025 were retrospectively analyzed.

Patients with a discharge diagnosis of heart failure (ICD-10 code I50) were identified through the electronic medical record (EMR) system. Eligible participants completed post-discharge follow-up at the Cardiac Rehabilitation Outpatient Clinic.

Inclusion Criteria: (1) Diagnosis of chronic heart failure (CHF) according to the 2018 Chinese Guidelines for the Diagnosis and Treatment of Heart Failure [5]. (2) New York Heart Association (NYHA) Functional Class II-IV. (3) Age ranging from 30 to 85 years. (4) Participation in the center’s standard post-discharge cardiac rehabilitation and follow-up program.

Exclusion Criteria: (1) Uncontrolled hypertension (systolic blood pressure >180 mmHg and/or diastolic blood pressure >110 mmHg). (2) Acute exacerbation phase of comorbidities that significantly impair cardiopulmonary function or prevent home-based exercise. (3) Severe non-cardiac organ dysfunction (hepatic, renal, or cerebral). (4) Malignancy. (5) Metabolic, infectious, or orthopedic-rheumatological diseases requiring hormone or chemotherapy. (6) Irregular medication use after discharge. (7) Substance abuse or cognitive impairment. (8) History of formal cardiopulmonary rehabilitation or significant non-cardiac conditions affecting survival during the study period. (9) Incomplete clinical records, or missing key baseline or 6-month follow-up assessment data.

### 2.2. Baseline Data and Routine In-Hospital Assessments

Baseline demographic, clinical, and laboratory data were extracted from Electronic Medical Records (EMR), including age, sex, body mass index (BMI), smoking history, comorbidities (diabetes mellitus, atrial fibrillation, and COPD), and serum creatinine. CHF severity was assessed using the New York Heart Association (NYHA) functional classification. Phenotypes were defined by left ventricular ejection fraction (LVEF) and categorized into heart failure with reduced (HFrEF, ≤40%), mildly reduced (HFmrEF, 41–49%), and preserved (HFpEF, ≥50%) ejection fraction. Primary CHF etiologies included ischemic cardiomyopathy, hypertensive heart disease, dilated cardiomyopathy (DCM), rheumatic valvular heart disease, and others. Furthermore, physical frailty status at discharge was retrospectively assessed using the validated Fried Frailty Phenotype, based on objective in-hospital data (handgrip strength and 6 min walk distance) and progress notes. Patients were classified as normal (0 criteria), pre-frail (1–2 criteria), or frail (≥3 criteria). 

All patients received guideline-directed medical therapy (GDMT) during hospitalization and follow-up, including diuretics, beta-blockers, angiotensin-converting enzyme inhibitors (ACEI), angiotensin II receptor blockers (ARB), angiotensin receptor-neprilysin inhibitors (ARNI), mineralocorticoid receptor antagonists (MRA), and sodium-glucose cotransporter 2 (SGLT-2) inhibitors. Concomitant therapies for glycemic and blood pressure control were maintained post-discharge.

The following standard inpatient cardiac rehabilitation assessments were retrieved:

(1) Exercise Capacity: Assessed via the 6-Minute Walk Test (6MWT) and handgrip strength. The 6MWT was conducted along a 30-meter flat, obstacle-free corridor, with patients walking at their maximum tolerable pace. Handgrip strength was measured using a dynamometer with the non-dominant hand in a standing position; patients squeezed maximally for 3–5 s, and the higher value from two trials (separated by a 15 s interval) was recorded.

(2) Cardiac Function: Included the last measurement of serum N-terminal pro-brain natriuretic peptide (NT-proBNP) and left ventricular ejection fraction (LVEF) prior to discharge.

(3) Quality of Life and Psychological Status: Evaluated using the Minnesota Living with Heart Failure Questionnaire (MLHFQ) [6], the Self-rating Somatic Symptom Scale-China (SSS-CN) [7], the Patient Health Questionnaire-9 (PHQ-9) [8], the Generalized Anxiety Disorder-7 (GAD-7) [9], and the Pittsburgh Sleep Quality Index (PSQI) [10]. Higher scores indicated poorer quality of life or psychological well-being.

### 2.3. Follow-Up Data, Retrospective Grouping and Study Outcomes

Data Retrieval and Follow-Up Management: At the 6-month follow-up, data from routine repeated clinical evaluations were retrieved from the outpatient database, including body weight, 6MWT distance, handgrip strength, NT-proBNP, LVEF, and the relevant questionnaires (MLHFQ, SSS-CN, PHQ-9, GAD-7, and PSQI). Prior to discharge, all included patients had received standardized technical instruction and education on pursed-lip diaphragmatic breathing (PLDB) from the cardiac rehabilitation team, as documented in the EMR. As part of the standard post-discharge care pathway, patients were enrolled in a WeChat-based cardiac rehabilitation management group, through which rehabilitation staff shared daily training videos to facilitate home-based practice.

Retrospective Grouping and Adherence Quantification: Patient adherence to the home-based PLDB protocol was evaluated retrospectively through a comprehensive triangulation of three primary data sources: (1) daily digital check-in records maintained within the managed WeChat rehabilitation group, (2) routine clinician-administered compliance inquiries documented in the EMR during the 6-month follow-up outpatient visit, and (3) patient self-reported training frequencies captured during structured follow-up telephone interviews. Based on the compiled training data from these multi-channel sources, patients were retrospectively assigned to one of two groups: 

(1) Respiratory Training Group: Achieved a consistent training intensity of ≥10 repetitions/set, 3 sets/day, for ≥3 days/week. 

(2) Control Group: Did not meet the aforementioned training threshold during the 6-month follow-up period.

Hierarchy and Definition of Study Outcomes: (1) Primary Outcome: Defined as the clinical prognosis, specifically the time to the first occurrence of Major Adverse Cardiovascular Events (MACE). MACE was a composite endpoint comprising rehospitalization for acute heart failure, malignant arrhythmias, acute myocardial infarction, and cardiac death. For this composite analysis, a time-to-first-event approach was utilized, where each patient contributed only once. Sequential clinical events occurring during a single continuous hospital admission (e.g., a heart failure rehospitalization subsequently leading to in-hospital cardiac death) were counted as a single MACE event. All-cause rehospitalization (defined as any hospital stay exceeding 24 h) was tracked and verified alongside MACE through WeChat interactions, telephone interviews, and the EMR tracking system. (2) Key Secondary Endpoints: Based on clinical relevance and data completeness, 6MWT distance, LVEF, and MLHFQ score were designated as the key continuous secondary endpoints to evaluate longitudinal changes in functional capacity, cardiac function, and quality of life, respectively. The MLHFQ score was prioritized as the sole key secondary questionnaire due to its disease-specific relevance to heart failure clinical status. Notably, serum NT-proBNP was a priori excluded from the key secondary inferential framework due to its high post-intervention missing rate (~40%) and a missing not at random (MNAR) mechanism; this pre-specified exclusion was implemented to prevent potential selection bias and severe statistical power loss in subsequent multivariate adjusted models. (3) Exploratory Mechanistic Endpoints: Generic psychological and sleep-related scales (GAD-7, PHQ-9, PSQI, SSS-CN) and handgrip strength were designated as exploratory mechanistic endpoints to investigate auxiliary treatment effects.

### 2.4. PLDB Training Protocol

**Figure 1:**
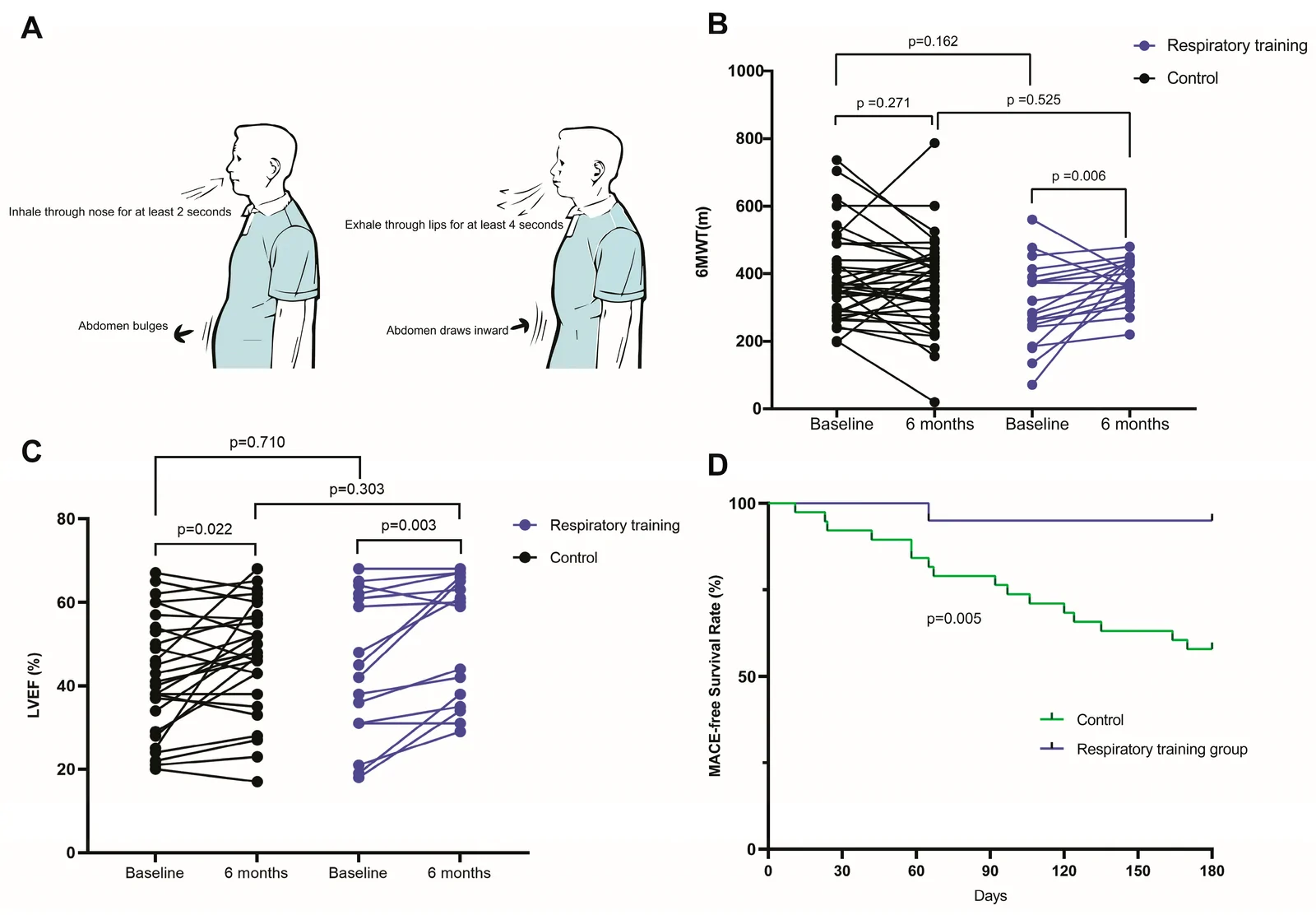
Comparison of PLDB mechanics, functional capacity, cardiac function, and clinical outcomes between the two groups. (**A**) Schematic diagram of PLDB movements. (**B**) Scatter plots comparing 6MWT distance before and after intervention between the two groups. (**C**) Scatter plots comparing LVEF before and after intervention between the two groups. (**D**) Kaplan–Meier survival analysis for MACE-free survival rate between the two groups. Abbreviations: PLDB, Pursed-Lip Diaphragmatic Breathing; 6MWT, 6-Minute Walk Test; LVEF, Left Ventricular Ejection Fraction; MACE, Major Adverse Cardiovascular Events.

Pursed-lip Diaphragmatic Breathing (PLDB) was performed following a standardized clinical protocol [11]. This technique emphasizes the coordination of diaphragmatic engagement and controlled expiratory resistance (Figure 1A).

The specific steps were as follows: (1) Preparation: Patients were instructed to assume either a seated or recumbent position with relaxed neck and shoulder muscles. (2) Diaphragmatic Inhalation: Patients inhaled deeply through the nose for at least 2 s, allowing the abdomen to rise significantly while minimizing chest movement. (3) Pursed-lip Exhalation: Patients exhaled slowly through pursed lips (in a “kissing” or whistling position) for at least 4 s, with the abdomen actively contracted inward as much as possible during exhalation. An exhalation-to-inhalation ratio of approximately 2:1 was used. (4) Frequency and Intensity: Patients were instructed to perform 10–15 cycles per session, with a total target of at least 30 cycles daily.

### 2.5. Statistical Analysis

Descriptive Statistics and Univariate Comparisons: Continuous variables were assessed for normality using the Shapiro–Wilk test. Normally distributed data were expressed as mean ± standard deviation (SD); between-group comparisons were analyzed using the independent Student’s *t*-test, and within-group comparisons before and after the intervention were evaluated using the paired *t*-test. For variables that did not follow a normal distribution at any time point, data were expressed as median (P25, P75) for consistency; between-group comparisons were analyzed using the Mann–Whitney U test, and within-group comparisons were evaluated using the Wilcoxon signed-rank test. Baseline ordinal variables (NYHA functional class and frailty status) were compared using the Mann–Whitney U test, and nominal categorical variables were analyzed using the chi-squared or Fisher’s exact test, as appropriate.

Time-to-Event Analyses: Follow-up time was defined from hospital discharge to the first MACE or the 6-month study endpoint, with event-free patients censored at their last visit. MACE-free survival was estimated using the Kaplan–Meier method and compared via the log-rank test. Univariate Cox proportional hazards regression was performed to calculate unadjusted hazard ratios (HRs) and 95% confidence intervals (CIs) for all prespecified baseline characteristics. For the multivariable model, covariate selection was guided by clinical relevance and biological plausibility rather than univariate *p* values. Given the limited number of observed MACEs (*n* = 17), the adjusted model was prespecified to include only treatment assignment and baseline LVEF to strictly adhere to the events-per-variable (EPV) principle and minimize overfitting. Baseline LVEF was retained as an a priori covariate per current heart failure guidelines [12,13], whereas other clinically relevant markers (e.g., MLHFQ score) were restricted to univariate screening to preserve model parsimony. All survival analyses utilized complete-case data.

Secondary Outcomes Analysis: Continuous secondary outcomes were analyzed hierarchically based on clinical relevance and data completeness. Key continuous secondary endpoints (6WT distance, LVEF, and MLHFQ score) were evaluated using analysis of covariance (ANCOVA) adjusted for their respective baseline values. To address violations of normality assumptions or to ensure robust inference for these key secondary metrics, bootstrap resampling (1000 iterations) was applied to construct 95% bias-corrected and accelerated confidence intervals (95% BCa CI). Psychological and sleep-related scales (GAD-7, PHQ-9, PSQI, SSS-CN) and handgrip strength were designated as exploratory mechanistic endpoints; between-group comparisons for these metrics were reported descriptively or using unadjusted tests, with explicit acknowledgment of limited statistical power and unadjusted multiplicity. All secondary analyses were pre-specified and interpreted in the context of the primary MACE outcome. 

Complete-Case Analyses, Handling of Missing Data: Post-intervention LVEF and NT-proBNP had missing rates of approximately 25.9% and 39.6%, respectively, with distinct underlying mechanisms: LVEF missingness was primarily logistical (satisfying MAR), whereas NT-proBNP missingness was driven by clinical and test-related factors (indicating MNAR). Given these heterogeneous patterns, complete-case analyses were conducted separately for each biomarker to maximize valid data utilization and preserve statistical power. For LVEF, the primary analysis employed a complete-case bootstrap ANCOVA to reflect directly observed clinical trends, with multiple imputation (generating five datasets) reserved for sensitivity analysis due to MI-induced variance inflation and inconsistent estimates (Supplementary Table S1). Conversely, to avoid biased estimates associated with applying MI to MNAR data, NT-proBNP was excluded from all multivariable models (including Cox regression and ANCOVA). All analyses for NT-proBNP were performed strictly within a complete-case framework using non-parametric descriptive methods, without imputation.

All statistical tests were two-tailed, and a *p* value < 0.05 was considered statistically significant. Statistical analyses, including the MI procedure, were conducted using SPSS version 22.0 (IBM Corp., Armonk, NY, USA), and statistical graphs were generated using GraphPad Prism version 9.5 (GraphPad Software, San Diego, CA, USA).

### 2.6. Patient and Public Involvement Statement

Neither Patient and Public Involvement Patients nor the public were involved in the design, conduct, reporting, or dissemination plans of this research.

## 3. Results

### 3.1. Data Completeness and Participant Flow

A total of 61 patients initially met the diagnostic criteria for CHF. Following screening, three patients were excluded: one due to a severe intra-hospital infection, one owing to a subsequent diagnosis of systemic lupus erythematosus, and one who discontinued medication post-discharge. Consequently, 58 patients formally enrolled and completed the 6-month follow-up. Based on documented 6-month post-discharge adherence, patients were retrospectively assigned to either the Respiratory Training Group (*n* = 20) or the Control Group (*n* = 38) (Supplementary Figure S1). As detailed in Table 1, baseline characteristics did not differ significantly between the two groups regarding age, sex, BMI, smoking history, CHF phenotypes, primary etiologies, serum creatinine levels, comorbidities (diabetes mellitus, atrial fibrillation, and COPD), and medications (all *p* > 0.05). Significant baseline differences were present only in the NYHA functional class (*p* = 0.041) and frailty status (*p* = 0.031). The Respiratory Training Group contained a higher proportion of NYHA class III patients (70.0% vs. 50.0%), whereas the Control Group exhibited a higher prevalence of baseline physical frailty (76.3% vs. 50.0%).

### 3.2. Effects on Exercise Capacity and Cardiac Function

Baseline 6MWT distance and handgrip strength did not differ significantly between groups (p=0.162 and p=0.227, respectively; Table 1). At 6 months, 6MWT distance in the Respiratory Training Group increased from 302.00 (243.60, 408.15) m to 386.00 (339.50, 439.00) m (Z=2.763,p=0.006; Figure 1B), and handgrip strength increased from 21.53 ± 9.32 kg to 25.60 ± 7.32 kg (t=2.131,p=0.046). No significant within-group changes in 6MWT distance (p=0.271) or handgrip strength (p=0.373) occurred in the Control Group. In the unadjusted follow-up analysis, hand-grip strength was significantly higher in the Respiratory Training Group than in the Control Group (t=−2.612, p=0.012, Table 2), whereas the between-group difference in 6MWT distance was not statistically significant (p=0.525, Table 2). Following baseline-adjusted bootstrap ANCOVA, the 6-month 6MWT distance was significantly greater in the Respiratory Training Group than in the Control Group (adjusted mean: 432.31 [SE 24.57] m vs. 356.14 [SE 17.96] m; adjusted difference: 76.16 m, 95% BCa CI: 7.64 to 141.17 m; bootstrap p=0.039; Supplementary Table S2).

Baseline LVEF and NT-proBNP levels did not differ significantly between groups (p=0.710 and p=0.366, respectively; Table 1). Due to missing follow-up records, paired analysis for LVEF included 16 patients in the Respiratory Training Group and 27 in the Control Group; for NT-proBNP, the analysis included 13 and 21 patients, respectively. Within-group LVEF increased significantly in both the Respiratory Training Group (44.25 ± 17.40% to 51.81 ± 14.89%, t=3.599,p=0.003) and the Control Group (42.44 ± 14.23% to 47.22 ± 13.34%, t=2.440, p=0.022; Figure 1C). The unadjusted between-group difference at 6 months was not statistically significant (p=0.303, Table 2). In the baseline-adjusted bootstrap ANCOVA, the 6-month adjusted mean LVEF did not differ significantly between the Respiratory Training Group and the Control Group (51.81% [SE 3.48%] vs. 47.22% [SE 2.68%]; adjusted difference: 4.59%, 95% BCa CI: −4.28% to 13.16%, bootstrap p=0.295; Supplementary Table S2). No statistically significant within-group changes or between-group differences were observed for NT-proBNP at 6 months (all p>0.05, Table 2).

Multiple imputation was conducted as a sensitivity analysis for missing follow-up echocardiography data (Supplementary Table S1). After imputation, the within-group LVEF change was statistically significant in the Respiratory Training Group (p=0.026) but not in the Control Group (p=0.341). Between-group comparisons at 6 months remained not statistically significant.

### 3.3. Improvements in Quality of Life and Psychological Indicators

Baseline MLHFQ, GAD-7, PHQ-9, PSQI, and SSS-CN scores did not differ significantly between groups (all p>0.05, Table 3). At 6 months, scores in the Respiratory Training Group decreased significantly for MLHFQ (40.00 ± 15.03 to 18.75 ± 13.47, t=−4.803,p<0.001), GAD-7 (2.00 [1.00, 5.00] to 0.50 [0, 2.75], Z=−2.580,p=0.010), and SSS-CN (36.00 [31.25, 39.00] to 30.00 [27.00, 34.00], Z=−2.899,p=0.004; Table 3). Post-intervention MLHFQ and SSS-CN scores were significantly lower in the Respiratory Training Group than in the Control Group (MLHFQ: Z=3.434,p=0.001; SSS-CN: Z=2.331,p=0.020; Table 3). Following baseline-adjusted bootstrap ANCOVA, the 6-month adjusted mean MLHFQ score remained significantly lower in the Respiratory Training Group versus the Control Group (18.85 [SE 3.93] vs. 37.29 [SE 2.88]; adjusted difference: −18.44, 95% BCa CI: −27.71 to −9.79; bootstrap p=0.001; Supplementary Table S2).

### 3.4. Clinical Outcomes and Proportional Hazards Analysis for MACE-Free Survival

During the follow-up period, 3 rehospitalization events occurred in the Respiratory Training Group (1 due to recurrent decompensated heart failure), and 16 occurred in the Control Group (all 16 attributed to worsening heart failure). No cardiac deaths occurred in the Respiratory Training Group, and 1 occurred in the Control Group. This death occurred during the patient’s admission for worsening heart failure; under the time-to-first-event framework, this sequence was counted as a single MACE. The composite MACE rate was significantly lower in the Respiratory Training Group than in the Control Group (5.0% vs. 42.1%, *p* = 0.003; Table 4). The MACE-free survival rate was significantly higher in the Respiratory Training Group than in the Control Group (log-rank *p* = 0.005, Figure 1D).

In univariate Cox regression analysis, group assignment to the Respiratory Training Group was significantly associated with a lower risk of MACE (HR 0.097, 95% CI 0.013–0.729, *p* = 0.023). Higher baseline MLHFQ scores were significantly associated with a higher risk of MACE (HR 1.050, 95% CI 1.027–1.074, *p* < 0.001). NYHA class III and IV showed a non-significant trend toward higher risk compared with class II (HR 1.867, *p* = 0.552; HR 2.014, *p* = 0.517, respectively). Baseline LVEF, age, sex, atrial fibrillation, and other baseline characteristics did not demonstrate significant univariate associations (all *p* > 0.05; full results provided in Supplementary Table S3). The final multivariable Cox model included a total of 17 MACEs. Given the limited number of observed events, the model was prespecified to include only treatment assignment and baseline LVEF to approximate the EPV ≥10 guideline while minimizing overfitting. Despite its univariate association (*p* < 0.001), the MLHFQ score was excluded from the adjusted model to avoid over-adjustment by introducing a subjective patient-reported metric into a survival model with limited events. Baseline LVEF was selected as the objective clinical covariate. In the adjusted model, PLDB training remained independently associated with a reduced risk of MACE (HR 0.103, 95% CI 0.014–0.779, *p* = 0.028). Baseline LVEF did not show a significant association with outcomes in the adjusted model (HR 1.007, 95% CI 0.974–1.041, *p* = 0.693) (Table 5).

## 4. Discussion

This retrospective study demonstrates that high adherence to a 6-month, lifestyle-integrated PLDB program is significantly associated with improved exercise capacity, reduced somatic and psychological symptoms, and a lower incidence of MACE in CHF patients. The baseline-adjusted between-group difference in 6MWT distance was 76.16 m, which substantially exceeds the widely accepted minimal clinically important difference (MCID) threshold of 30–35 m for HF populations [14,15]. This confirms that the observed functional improvement is not only statistically significant but also clinically meaningful for patients, suggesting that sustained adherence to this lifestyle-integrated breathing modality correlates with clinically meaningful improvements in exercise tolerance, even within a non-supervised, home-based environment.

Exertional dyspnea in CHF arises from a maladaptive cycle of peripheral/respiratory muscle atrophy, pulmonary congestion, and neurohormonal overactivation [16,17,18]. Together, these factors establish a vicious cycle of “activity limitation–physical capacity decline–symptom exacerbation”. Targeted inspiratory muscle training (IMT) or respiratory rehabilitation prolongs inspiratory duration, reduces cardiac afterload, increases cardiac output, and enhances both respiratory muscle function and lung capacity [19]. Although recent guidelines have integrated such training into CHF rehabilitation protocols [20,21], specific implementation details remain scarce, rendering the optimization of respiratory training a key research focus. Sadek et al. conducted a meta-analysis that identified the optimal IMT protocol: six sessions per week at 60% maximal inspiratory pressure for 30 min over 12 weeks, which maximally improved inspiratory strength, 6MWT distance, and dyspnea [22]. This approach necessitates specialized equipment and professional supervision, limiting its feasibility in remote or resource-constrained settings. Furthermore, frail patients may struggle to achieve target intensities, increasing the risk of adverse events. Kawauchi et al. noted that low-to-moderate intensity IMT similarly improves muscle strength and exercise tolerance but shows inconsistent effects on heart failure symptoms and NYHA classification [23]. Our study expands these insights by validating a device-free, low-intensity, home-based strategy.

Analogous to “qi sinking to the dantian” in Tai Chi, PLDB regulates psychophysiological states via slow, rhythmic breathing. The observed biological and psychological benefits in the adherent cohort can be mapped to several synergistic pathways: (1) Pursed-lip exhalation increases intraluminal airway pressure, mitigating small airway collapse, while diaphragmatic breathing strengthens diaphragmatic excursion and reduces respiratory resistance [24,25]. These effects synergistically reduce cardiac preload. Furthermore, diaphragmatic breathing promotes coordinated abdominal muscle contraction, optimizing the recruitment of respiratory muscles (e.g., internal intercostals and the diaphragm) that typically become inefficient during pulmonary congestion. Improved ventilation optimizes skeletal muscle aerobic metabolism, promotes protein synthesis, and enhances exercise tolerance [26]. Together, these factors ultimately help break the vicious cycle of “activity limitation–physical capacity decline–symptom exacerbation” (Supplementary Figure S2). (2) Slow–deep breathing enhances parasympathetic tone and attenuates sympathetic overactivity, improving heart rate variability and reducing norepinephrine levels [27,28,29]. This decreases myocardial oxygen consumption and arrhythmic risk. The subsequent inhibition of the renin–angiotensin–aldosterone system (RAAS) reduces the release of cardiotoxic mediators [30]. (3) These physiological gains alleviate dyspnea and fatigue, fostering patient autonomy and reducing anxiety, as consistently demonstrated across multiple clinical studies [31,32]. However, we must explicitly acknowledge that these physiological and neurohormonal pathways—including autonomic balance, HRV parameters, norepinephrine levels, and RAAS activation—were not directly measured via biomarkers or device-based diagnostics in the present study. Consequently, while these physiological mechanisms are grounded in the established clinical literature, their direct contribution to the outcomes of our specific cohort remains speculative and must be interpreted as hypothesized frameworks rather than verified observational facts.

A critical methodological distinction of this study is that all patients received identical, standardized PLDB instruction prior to discharge; thus, group allocation was retrospectively determined by 6-month adherence rather than randomization. This design positions our findings as a real-world prognostic association rather than a demonstration of controlled efficacy, inherently introducing healthy-adherer bias and potential confounding. However, baseline data directly challenge the assumption that high adherence merely reflects milder baseline disease. Baseline LVEF was comparable between groups (*p* = 0.710), and this comparability persisted even within the complete-case subcohort (Training: *n* = 16; Control: *n* = 27). Notably, the training group actually comprised a significantly higher proportion of NYHA class III patients (70.0% vs. 50.0%, *p* = 0.041), indicating a more advanced baseline clinical stage. This divergence suggests that sustained adherence was not simply a byproduct of healthier baseline status, but may instead be driven by unmeasured psychosocial or socioeconomic factors, such as heightened health literacy, stronger motivation for symptom relief, or greater educational attainment. Nevertheless, residual confounding from the healthy-adherer effect cannot be entirely excluded, as highly adherent individuals may concurrently engage in other beneficial health behaviors (e.g., strict medication/diet adherence, fluid management, and regular follow-ups) that independently improve prognosis.

Furthermore, the risk of reverse causation represents a profound structural confounder in retrospective survival analyses, as patients who developed worsening heart failure or experienced early rehospitalization episodes within the first 6 months were frequently rendered physically unable to remain adherent to the training criteria, automatically allocating them to the Control Group and mathematically compounding the apparent incidence of MACE in that cohort. To evaluate the impact of this automatic classification bias, we conducted a lagged sensitivity analysis by sequentially truncating early MACEs (Supplementary Table S4). Even after the complete exclusion of patients who experienced MACE within the initial 2 months of follow-up—which mathematically reduced the total event count to a highly sparse 11 events—the Kaplan–Meier survival curves still demonstrated a statistically significant difference between the two cohorts (Log-Rank *p* = 0.031). Although the corresponding multivariable Cox model exhibited expected statistical underpowering due to this extreme event reduction, resulting in a wider confidence interval and a marginal *p*-value (adjusted HR = 0.146, *p* = 0.067), the absolute magnitude and directional alignment of the hazard ratio remained highly consistent with the primary analysis (unadjusted HR shifted minorly from 0.097 to 0.144). Given these overlapping biases and the demonstrated stability of the lagged estimates, the lower hazard of MACE observed in the Respiratory Training Group must be interpreted strictly as an epidemiological association with adherence, rather than a direct causal consequence of the PLDB intervention.

Contemporary cardiac rehabilitation is increasingly shifting from rigid, structured regimens toward fragmented, lifestyle-integrated physical activity. Emerging evidence validates the cardiovascular benefits of “exercise snacks” and incidental physical activity (IPA) for patients unable to sustain prolonged structured exercise [33,34]. Our “lifestyle-based active training” model aligns with this paradigm, offering distinct pragmatic advantages: superior safety (mitigating exercise-induced ischemia), broad clinical applicability (feasible even in NYHA class IV), and high cost-effectiveness (eliminating equipment and facility dependencies). By embedding flexible, low-intensity breathing exercises into daily routines (e.g., post-awakening or post-meal), PLDB enhances long-term behavioral sustainability without imposing the spatial or intensity constraints of conventional rehabilitation.

The unchanged NT-proBNP levels despite improvements in functional capacity and clinical outcomes warrant careful interpretation. This apparent discrepancy likely stems from two interrelated factors. First, the high missing rate at follow-up (~40%) substantially reduced statistical power, increasing the risk of a type II error and limiting our ability to detect modest biomarker changes. Meanwhile, the missing NT-proBNP data follow an MNAR pattern, which precludes multiple imputation for sensitivity analysis, as described in the Methods. Second, this dissociation aligns with established pathophysiological principles in heart failure rehabilitation: functional metrics like the 6MWT and symptomatic scores reflect rapid peripheral adaptations (e.g., skeletal/respiratory muscle efficiency and autonomic modulation), whereas NT-proBNP is a stringent marker of central hemodynamic wall stress and neurohormonal activation, which often require longer durations or more intensive hemodynamic unloading to normalize [16,17,19]. Thus, the absence of significant NT-proBNP reduction does not negate the observed clinical benefits but rather highlights the compartmentalized nature of short-term rehabilitation responses. Future studies with complete biomarker follow-up and extended observation periods are needed to clarify the temporal relationship between functional recovery and neurohormonal remodeling. Similarly, LVEF improvements warrant careful contextualization given 25.9% missing echocardiographic data. Our primary cardiac conclusions are anchored in complete-case analysis of directly measured data, which revealed significant within-group LVEF improvement in both arms—consistent with expected myocardial recovery under sustained GDMT in the control group. Multiple imputation, while methodologically sound for handling missingness, incorporates estimation uncertainty that can inflate standard errors and attenuate marginal significance in smaller cohorts, while inherently regressing estimates toward the mean. Therefore, cardiac function outcomes should be interpreted primarily through the lens of measured complete-case data, with imputation serving strictly as a supplementary robustness check.

Several limitations warrant transparent acknowledgment. First, the relatively small single-center sample (*n* = 58) limits the generalizability of our findings and prohibits robust subgroup analyses stratified by heart failure etiology or LVEF phenotypes. Second, the survival analysis was constrained by a low number of MACEs (*n* = 17 over 6 months). The initial multivariable Cox model violated the EPV principle by incorporating multiple covariates with limited endpoint events, which could cause model overfitting, statistical instability, and biased effect estimates. Although we revised the model to include only two covariates (group allocation and baseline LVEF), the small event count still restricts the precision of adjusted outcomes. The wide 95% CI (0.014–0.779) reflects the inherent statistical imprecision associated with limited event counts, and the point estimate should be interpreted with caution. Third, exercise tolerance was exclusively evaluated using the 6MWT. While the 6MWT is a highly accessible and validated practical tool, it remains an indirect, submaximal proxy for true cardiorespiratory fitness and can be influenced by subjective patient motivation. Cardiopulmonary exercise testing (CPET) remains the clinical gold standard for functional profiling in heart failure, providing objective, immutable parameters such as peak oxygen consumption (peak *VO*_2_), carbon dioxide production, respiratory exchange ratio, and the ventilatory efficiency slope (*VE*/*VCO*_2_ slope). These parameters allow for precise differentiation between cardiac, pulmonary, and peripheral skeletal muscle limitations. The absence of baseline and follow-up CPET data prevents us from identifying the exact metabolic or breath-by-breath physiological adaptations associated with PLDB adherence, representing a major missing element in our mechanistic evaluation. Additionally, we did not measure autonomic and neurohormonal biomarkers (e.g., HRV, catecholamines, RAAS components), rendering the proposed mechanistic pathways speculative. Fourth, adherence was retrospectively determined via WeChat records, telephone follow-ups, and outpatient interviews without standardized daily training logs, which may introduce recall and social desirability biases and limits the accurate quantification of home-based training quality. Finally, the open-label design and 6-month follow-up duration restrict long-term prognostic evaluation and causal inference. Future multicenter, prospective randomized trials incorporating comprehensive CPET, autonomic/neurohormonal monitoring, and extended follow-up are essential to validate the causal relationship between PLDB adherence and hard cardiovascular outcomes.

## Figures and Tables

**Table 1:** Baseline demographic characteristics of respiratory training and control groups.

Characteristics		Respiratory Training Group (*n* = 20)	Control Group (*n* = 38)	*p* Value
Age, years		66.85 ± 12.01	70.68 ± 10.09	0.203 ^a^
BMI, kg/m^2^		22.95 (20.90, 25.92)	22.45 (19.57, 24.92)	0.152 ^b^
Male/female, n		16/4	23/15	0.155 ^d^
Smoking, *n* (%)		7 (35.0)	15 (39.5)	0.739 ^c^
Diabetes Mellitus, *n* (%)		9 (45.0)	9 (23.7)	0.095 ^c^
Atrial Fibrillation, *n* (%)		3 (15.0)	14 (36.8)	0.082 ^c^
COPD, *n* (%)		1 (5.0)	0 (0.0)	0.345 ^d^
6MWT, m		302.00 (243.60, 408.15)	360.00 (285.00, 452.25)	0.162 ^b^
Hand grip strength, Kg		21.53 ± 9.32	18.98 ± 6.43	0.227 ^a^
LVEF, %		44.90 ± 17.03	46.54 ± 15.11 * (*n* = 37)	0.710 ^a^
NT-proBNP, pg/mL		2550.00 (473.00, 5220.00) * (*n* = 17)	2360.00 (665.00, 3190.00) * (*n* = 37)	0.366 ^b^
Serum Creatinine, μmol/L		80.00 (56.75, 104.50)	85.00 (70.00, 107.75)	0.303 ^b^
NYHA Class				0.041 ^b^
Class II, *n* (%)		3 (15.0)	16 (42.1)	
Class III, *n* (%)		14 (70.0)	19 (50.0)	
Class IV, *n* (%)		3 (15.0)	3 (7.9)	
Phenotypes				0.650 ^c^
HFpEF, *n* (%)		7 (35.0)	18 (47.4)	
HFmrEF, *n* (%)		5 (25.0)	7 (18.4)	
HFrEF, *n* (%)		8 (40.0)	13 (34.2)	
Primary etiologies				0.332 ^d^
Ischemic, *n* (%)		12 (60.0)	16 (42.1)	
Hypertensive, *n* (%)		1 (5.0)	8 (21.1)	
DCM, *n* (%)		2 (10.0)	8 (21.1)	
Rheumatic, *n* (%)		3 (15.0)	3 (7.9)	
Other, *n* (%)		2 (10.0)	3 (7.9)	
Frailty Status				0.031 ^b^
Normal, *n* (%)		3 (15.0)	1 (2.6)	
Pre-frail, *n* (%)		7 (35.0)	8 (21.1)	
Frail, *n* (%)		10 (50.0)	29 (76.3)	
Medication *n* (%)	Diuretics	15 (75.0)	30 (78.9)	0.732 ^c^
	ARNI/ACEI/ARB	18 (90.0)	32 (84.2)	0.543 ^c^
	β-blocker	17 (85.0)	23 (60.5)	0.056 ^c^
	SGLT-2 inhibitor	16 (80.0)	28 (73.7)	0.593 ^c^

Abbreviations: NYHA, New York Heart Association; HFpEF, Heart Failure with preserved Ejection Fraction; HFmrEF, Heart Failure with mildly reduced Ejection Fraction; HFrEF, Heart Failure with reduced Ejection Fraction; COPD, Chronic Obstructive Pulmonary Disease; DCM, Dilated Cardiomyopathy; BMI, Body Mass Index; ARNI, Angiotensin Receptor–Neprilysin Inhibitor; ACEI, Angiotensin-Converting Enzyme Inhibitor; ARB, Angiotensin II Receptor Blocker; SGLT-2, Sodium-Glucose Cotransporter 2. Note: Data are presented as mean ± standard deviation (SD) for normally distributed continuous variables, median (P25, P75) for non-normally distributed continuous variables. * Due to missing baseline clinical measurements for certain patients, the actual sample sizes (*n*) for LVEF and NT-proBNP are slightly smaller than the total enrolled population. The actual sample size for each specific variable is indicated in the table. ^a^ *p* values reported based on independent *t*-test. ^b^ *p* values reported based on Mann–Whitney U test. ^c^ *p* values reported based on Pearson’s Chi-square test. ^d^ *p* values reported based on Fisher exact test.

**Table 2:** Exercise tolerance and cardiac function between the two groups.

Characteristics	Respiratory Training Group			Control Group			Baseline Compared *p* Value (*t*/*Z*)	Between-Group Compared *p* Value (*t*/*Z*)
	Baseline	6 Months	Within-Group Difference, *p* Value (*t*/*Z*)	Baseline	6 Months	Within-Group Difference, *p* Value (*t*/*Z*)		
6MWT, m	302.00 (243.60, 408.15) (*n* = 20)	386.00 (339.50, 439.00) (*n* = 20)	0.006 ^f^ (*Z* = 2.763)	360.00 (285.00, 452.25) (*n* = 37)	391.00 (283.00, 442.00) (*n* = 37)	0.271 ^f^ (*Z* = 1.100)	0.162 ^b^ (*Z* = 1.399)	0.525 ^b^ (*Z* = 0.636)
Hand grip strength, Kg	21.53 ± 9.32 (*n* = 20)	25.60 ± 7.32 (*n* = 20)	0.046 ^e^ (*t* = 2.131)	18.98 ± 6.43 (*n* = 37)	20.07 ± 7.77 (*n* = 37)	0.373 ^e^ (*t* = 0.903)	0.227 ^a^ (*t* = −1.221)	0.012 ^a^ (*t* = −2.612)
LVEF, %	44.25 ± 17.40 (*n* = 16)	51.81 ± 14.89 (*n* = 16)	0.003 ^e^ (*t* = 3.599)	42.44 ± 14.23 (*n* = 27)	47.22 ± 13.34 (*n* = 27)	0.022 ^e^ (*t* = 2.440)	0.710 ^a^ (*t* = 0.374)	0.303 ^a^ (*t* = −1.004)
NT-proBNP, pg/mL	2550.00 (473.00, 5220.00) (*n* = 13)	571.00 (207.00, 2975.00) (*n* = 13)	0.087 ^f^ (*Z* = −1.712)	2360.00 (665.00, 3190.00) (*n* = 21)	1040.00 (320.00, 4315.00) (*n* = 21)	0.566 ^f^ (*Z* = −0.574)	0.366 ^b^ (*Z* = −0.903)	0.446 ^b^ (*Z* = −0.762)

Abbreviations: 6MWT, 6-Minute Walk Test; LVEF, Left Ventricular Ejection Fraction; NT-proBNP, N-terminal pro-brain natriuretic peptide; SD, Standard Deviation. Note: Data are presented as mean ± SD or median (P25, P75) based on the original available records. This table presents complete-case paired analyses, evaluated independently for each parameter among patients with complete records at both time points. The specific sample size for each analysis is indicated in parentheses below the respective data. ^a^ *p* values reported based on independent *t*-test. ^b^ *p* values reported based on Mann–Whitney U test. ^e^ *p* values for within-group changes reported based on paired Student’s *t*-test. ^f^ *p* values reported based on Wilcoxon’s signed-rank test.

**Table 3:** Quality of life and psychological indicators between the two groups.

Characteristics	Respiratory Training Group (*n* = 20)			Control Group (*n* = 37) *			Baseline Compared *p* value (*Z*)	Between-Group Compared *p* Value (*Z*)
	Baseline	6 Months	Within-Group Difference, *p* Value (*t*/*Z*)	Baseline	6 Months	Within-Group Difference, *p* Value (*t*/*Z*)		
MLHFQ, score	40.00 ± 15.03	18.75 ± 13.47	<0.001 ^e^ (*t* = −4.803)	39.00 (28.50, 58.00)	38.00 (19.00, 53.50)	0.181 ^f^ (*Z* = −1.336)	0.847 ^b^ (*Z* = −0.192)	0.001 ^b^ (*Z* = 3.434)
GAD7, score	2.00 (1.00, 5.00)	0.50 (0, 2.75)	0.010 ^f^ (*Z* = −2.580)	3.00 (1.00, 4.00)	2.00 (0.00, 4.50)	0.170 ^f^ (*Z* = −1.371)	0.826 ^b^ (*Z* = −0.220)	0.061 ^b^ (*Z* = 1.871)
PHQ9, score	6.00 (3.00, 9.75)	4.00 (2.00, 6.00)	0.111 ^f^ (*Z* = −1.594)	5.00 (4.00, 10.00)	5.00 (3.00, 9.50)	0.629 ^f^ (*Z* = −0.483)	0.906 ^b^ (*Z* = −0.118)	0.230 ^b^ (*Z* = 1.200)
PSQI, score	8.00 (4.25, 10.75)	5.55 (4.00,10.00)	0.468 ^f^ (*Z* = −0.725)	8.57 ± 3.76	8.78 ± 3.73	0.754 ^e^ (*t* = 0.316)	0.529 ^b^ (*Z* = −0.630)	0.085 ^b^ (*Z* = 1.722)
SSS-CN, score	36.00 (31.25, 39.00)	30.00 (27.00, 34.00)	0.004 ^f^ (*Z* = −2.899)	36.00 (30.00, 38.00)	35.00 (30.00, 38.50)	0.681 ^f^ (*Z* = −0.411)	0.847 ^b^ (*Z* = −0.193)	0.020 ^b^ (*Z* = 2.331)

Abbreviations: MLHFQ, Minnesota Living with Heart Failure Questionnaire; PHQ-9, Patient Health Questionnaire-9; GAD-7, Generalized Anxiety Disorder-7; PSQI, Pittsburgh Sleep Quality Index; SSS-CN, Self-Rating Somatic Symptom Scale-China; SD, Standard Deviation. Note: * One patient in the Control Group died before the 6-month evaluation and was excluded from this analysis; data are presented as mean ± SD or median (P25, P75) based on the original available records; ^b^ *p* values reported based on Mann–Whitney U test. ^e^ *p* values for within-group changes reported based on paired Student’s *t*-test. ^f^ *p* values reported based on Wilcoxon’s signed-rank test.

**Table 4:** The 6-month prognostic events between the two groups.

Groups	Re-Hospitalization (%)	Re-Hospitalization Due to Heart Failure (%)	Malignant Arrhythmia (%)	Acute Myocardial Infarction (%)	Cardiac Death (%)	Total MACE (%)
Control group (*n* = 38)	16 (42.1)	16 (42.1)	0	0	1 (2.6)	16 (42.1)
Respiratory training group (*n* = 20)	3 (15.0)	1 (5.0)	0	0	0	1 (5.0)
*p* value	0.076 ^d^					0.003 ^d^

Abbreviations: MACE, Major Adverse Cardiovascular Events. Note: ^d^ *p* values reported based on Fisher’s exact test.

**Table 5:** Proportional hazards regression analysis for 6-month MACE.

Characteristics		Univariate Analysis		Multivariate Analysis *	
		HR (95% CI)	*p* Value	HR (95% CI)	*p* Value
PLDB training (vs. Control)		0.097 (0.013–0.729)	0.023	0.103 (0.014–0.779)	0.028
Baseline LVEF (per 1% increase)		1.008 (0.977–1.041)	0.608	1.007 (0.974–1.041)	0.693
Age (per 1-year increase)		1.027 (0.981–1.075)	0.248	-	-
Gender (Male vs. Female)		0.673 (0.256–1.769)	0.422	-	-
Atrial Fibrillation (Yes vs. No)		1.889 (0.717–4.973)	0.198	-	-
MLHFQ score (per 1-point increase)		1.050 (1.027–1.074)	<0.001	-	-
NYHA Class	Class III vs. II	1.867 (0.239–14.589)	0.552	-	-
	Class IV vs. II	2.014 (0.242–16.736)	0.517	-	-

Abbreviations: CI, Confidence Interval; EPV, Events Per Variable; HR, Hazard Ratio; LVEF, Left Ventricular Ejection Fraction; MACE, Major Adverse Cardiovascular Events; MLHFQ, Minnesota Living with Heart Failure Questionnaire; NYHA, New York Heart Association; PLDB, Pursed-Lip Diaphragmatic Breathing. Note: * Covariate selection was restricted to treatment assignment and baseline LVEF to adhere to the EPV principle given the limited number of MACEs (*n* = 17), thereby minimizing the risk of overfitting. Regression analysis was performed for all baseline characteristics. Variables with *p* ≥ 0.20 or high collinearity with included metrics (e.g., age, comorbidities, functional capacity measures, and medications) are presented in Supplementary Table S3.

## Data Availability

The data underlying this article cannot be shared publicly due to the privacy of individuals that participated in the study. The data will be shared on reasonable request to the corresponding author.

## Supplementary Materials

The following supporting information can be downloaded at: https://www.mdpi.com/article/10.3390/healthcare14162489/s1, Table S1: Sensitivity analysis of LVEF: Comparison between complete-case analysis and multiple imputation analysis; Table S2: Between-Group Comparison of Core Secondary Outcomes at 6 Months Using Bootstrap ANCOVA; Table S3: Univariate Cox Proportional Hazards Regression Analysis for MACE Across Baseline Characteristics; Table S4: Sensitivity Analyses for MACE-Free Survival Excluding Early Events. Figure S1. Study design and flow chart. Figure S2. Mechanism of PLDB Training in Blocking the “Activity Limitation-Physical Capacity Decline-Symptom Exacerbation” Vicious Cycle. Caption: The green box denotes the inhibitory effects induced by PLDB training, the red box signifies the promoting effects, and the concentric circle diagram located in the lower part of the figure elucidates the “activity limitation-physical capacity decline-symptom exacerbation” vicious cycle in chronic heart failure (CHF). Abbreviations: PLDB, Pursed-lip Diaphragmatic Breathing. The figure was created with MedPeer (medpeer.cn).

## Abbreviations

The following abbreviations are used in this manuscript:

ACEI	Angiotensin-Converting Enzyme Inhibitors
ANCOVA	Analysis of Covariance
ARB	Angiotensin II Receptor Blockers
ARNI	Angiotensin Receptor-Neprilysin Inhibitors
BMI	Body Mass Index
CHF	Chronic Heart Failure
CIs	Confidence Intervals
CVD	Cardiovascular Disease
COPD	Chronic Obstructive Pulmonary Disease
DCM	Dilated Cardiomyopathy
EMR	Electronic Medical Record
EPV	Events-Per-Variable
GAD-7	Generalized Anxiety Disorder-7
GDMT	Guideline-Directed Medical Therapy
HFpEF	Heart Failure with preserved Ejection Fraction
HFmrEF	Heart Failure with mildly reduced Ejection Fraction
HFrEF	Heart Failure with reduced Ejection Fraction
HRs	Hazard Ratios
IMT	Inspiratory Muscle Training
IPA	Incidental Physical Activity
LVEF	Left Ventricular Ejection Fraction
MACE	Major Adverse Cardiovascular Events
MAR	Missing At Random
MCID	Minimal Clinically Important Difference
MNAR	Missing Not At Random
MI	Multiple Imputation
MLHFQ	Minnesota Living with Heart Failure Questionnaire
MRA	Mineralocorticoid Receptor Antagonists
6MWT	6-Minute Walk Test
NYHA	New York Heart Association
NT-proBNP	N-terminal pro-Brain Natriuretic Peptide
QoL	Quality of Life
PHQ-9	Patient Health Questionnaire-9
PLDB	Pursed-Lip Diaphragmatic Breathing
SD	Standard Deviation
SSS-CN	Self-rating Somatic Symptom Scale-China
SGLT-2	Sodium–Glucose Cotransporter 2
PSQI	Pittsburgh Sleep Quality Index
RAAS	Renin–Angiotensin–Aldosterone System
RTG	Respiratory Training Group

## References

[1] Perez-Moreno A.C., Jhund P.S., MacDonald M.R., Petrie M.C., Cleland J.G., Böhm M., van Veldhuisen D.J., Gullestad L., Wikstrand J., Kjekshus J. (2014). Fatigue as a predictor of outcome in patients with heart failure: Analysis of CORONA (Controlled Rosuvastatin Multinational Trial in Heart Failure). JACC Heart Fail..

[2] Fernandez-Rubio H., Becerro-De-Bengoa-Vallejo R., Rodríguez-Sanz D., Calvo-Lobo C., Vicente-Campos D., Chicharro J.L. (2020). Inspiratory Muscle Training in Patients with Heart Failure. J. Clin. Med..

[3] Yang Y., Wei L., Wang S., Ke L., Zhao H., Mao J., Li J., Mao Z. (2022). The effects of pursed lip breathing combined with diaphragmatic breathing on pulmonary function and exercise capacity in patients with COPD: A systematic review and meta-analysis. Physiother. Theory Pract..

[4] Azambuja A.D.C.M., Oliveira L.Z.D., Sbruzzi G. (2020). Inspiratory Muscle Training in Patients with Heart Failure: What Is New? Systematic Review and Meta-Analysis. Phys. Ther..

[5] Heart Failure Group (2018). Chinese guidelines for the diagnosis and treatment of heart failure 2018. Zhonghua Xin Xue Guan Bing Za Zhi.

[6] Heravi-Karimooi M., Bandari R., Eskandari S., Semnani S., Rejeh N., Montazeri A. (2025). Validation Study of the Iranian Version of Minnesota Living with Heart Failure Questionnaire (MLHF-Q): A Cross-Sectional Study. Health Sci. Rep..

[7] Jiang M., Zhang W., Su X., Gao C., Chen B., Feng Z., Mao J., Pu J. (2019). Identifying and measuring the severity of somatic symptom disorder using the Self-reported Somatic Symptom Scale-China (SSS-CN): A research protocol for a diagnostic study. BMJ Open.

[8] Kroenke K., Spitzer R.L., Williams J.B. (2001). The PHQ-9: Validity of a brief depression severity measure. J. Gen. Intern. Med..

[9] Spitzer R.L., Kroenke K., Williams J.B., Löwe B. (2006). A brief measure for assessing generalized anxiety disorder: The GAD-7. Arch. Intern. Med..

[10] Buysse D.J., Reynolds C.F., Monk T.H., Berman S.R., Kupfer D.J. (1989). The Pittsburgh Sleep Quality Index (PSQI): An instrument for psychiatric practice and research. Psychiatry Res..

[11] Vatwani A. (2019). Pursed Lip Breathing Exercise to Reduce Shortness of Breath. Arch. Phys. Med. Rehabil..

[12] McDonagh T.A., Metra M., Adamo M., Gardner R.S., Baumbach A., Böhm M., Burri H., Butler J., Čelutkienė J., Chioncel O. (2021). 2021 ESC Guidelines for the diagnosis and treatment of acute and chronic heart failure. Eur. Heart J..

[13] Heidenreich P.A., Bozkurt B., Aguilar D., Allen L.A., Byun J.J., Colvin M.M., Deswal A., Drazner M.H., Dunlay S.M., Evers L.R. (2022). 2022 AHA/ACC/HFSA Guideline for the Management of Heart Failure: A Report of the American College of Cardiology/American Heart Association Joint Committee on Clinical Practice Guidelines. Circulation.

[14] Bohannon R.W., Crouch R. (2017). Minimal clinically important difference for change in 6-minute walk test distance of adults with pathology: A systematic review. J. Eval. Clin. Pract..

[15] Singh S.J., Puhan M.A., Andrianopoulos V., Hernandes N.A., Mitchell K.E., Hill C.J., Lee A.L., Camillo C.A., Troosters T., Spruit M.A. (2014). An official systematic review of the European Respiratory Society/American Thoracic Society: Measurement properties of field walking tests in chronic respiratory disease. Eur. Respir. J..

[16] Craig J.C., Colburn T.D., Caldwell J.T., Hirai D.M., Tabuchi A., Baumfalk D.R., Behnke B.J., Ade C.J., Musch T.I., Poole D.C. (2019). Central and peripheral factors mechanistically linked to exercise intolerance in heart failure with reduced ejection fraction. Am. J. Physiol. Heart Circ. Physiol..

[17] Obokata M., Olson T.P., Reddy Y.N.V., Melenovsky V., Kane G.C., Borlaug B.A. (2018). Haemodynamics, dyspnoea, and pulmonary reserve in heart failure with preserved ejection fraction. Eur. Heart J..

[18] Nakagawa N.K., Diz M.A., Kawauchi T.S., de Andrade G.N., Umeda I.I.K., Murakami F.M., Oliveira-Maul J.P., Nascimento J.A., Nunes N., Takada J.Y. (2020). Risk factors for inspiratory muscle weakness in chronic heart failure. Respir. Care.

[19] Cahalin L.P., Arena R.A. (2015). Breathing exercises and inspiratory muscle training in heart failure. Heart Fail. Clin..

[20] Piepoli M.F., Conraads V., Corrà U., Dickstein K., Francis D.P., Jaarsma T., McMurray J., Pieske B., Piotrowicz E., Schmid J.-P. (2011). Exercise training in heart failure: From theory to practice. A consensus document of the Heart Failure Association and the European Association for Cardiovascular Prevention and Rehabilitation. Eur. J. Heart Fail..

[21] Shoemaker M.J., Dias K.J., Lefebvre K.M., Heick J.D., Collins S.M. (2020). Physical Therapist Clinical Practice Guideline for the Management of Individuals with Heart Failure. Phys. Ther..

[22] Sadek Z., Salami A., Joumaa W.H., Awada C., Ahmaidi S., Ramadan W. (2018). Best mode of inspiratory muscle training in heart failure patients: A systematic review and meta-analysis. Eur. J. Prev. Cardiol..

[23] Kawauchi T.S., Umeda I.I.K., Braga L.M., Mansur A.d.P., Rossi-Neto J.M., Sousa A.G.d.M.R., Hirata M.H., Cahalin L.P., Nakagawa N.K. (2017). Is there any benefit using low-intensity inspiratory and peripheral muscle training in heart failure? A randomized clinical trial. Clin. Res. Cardiol..

[24] Breslin E.H. (1992). The pattern of respiratory muscle recruitment during pursed-lip breathing. Chest.

[25] Yamaguti W.P., Claudino R.C., Neto A.P., Chammas M.C., Gomes A.C., Salge J.M., Moriya H.T., Cukier A., Carvalho C.R. (2012). Diaphragmatic Breathing Training Program Improves Abdominal Motion During Natural Breathing in Patients with Chronic Obstructive Pulmonary Disease: A Randomized Controlled Trial. Arch. Phys. Med. Rehabil..

[26] O’Halloran K.D. (2023). Pump up the volume: Breathing facilitates cardiac output during exercise by mechanical action. J. Physiol..

[27] Laborde S., Allen M.S., Borges U., Dosseville F., Hosang T., Iskra M., Mosley E., Salvotti C., Spolverato L., Zammit N. (2022). Effects of voluntary slow breathing on heart rate and heart rate variability: A systematic review and a meta-analysis. Neurosci. Biobehav. Rev..

[28] Lin I.M., Tai L.Y., Fan S.Y. (2014). Breathing at a rate of 5.5 breaths per minute with equal inhalation-to-exhalation ratio increases heart rate variability. Int. J. Psychophysiol..

[29] Jaenisch R.B., Quagliotto E., Chechi C., Calegari L., dos Santos F., Borghi-Silva A., Lago P.D. (2017). Respiratory Muscle Training Improves Chemoreflex Response, Heart Rate Variability, and Respiratory Mechanics in Rats with Heart Failure. Can. J. Cardiol..

[30] Triposkiadis F., Briasoulis A., Kitai T., Magouliotis D., Athanasiou T., Skoularigis J., Xanthopoulos A. (2024). The sympathetic nervous system in heart failure revisited. Heart Fail. Rev..

[31] Wang Z., Yu L., Song C., Cao H., Yang Z., Qiao S., Li X., Xie J. (2025). Effects of loaded deep breathing training combined with PERMA model in lung cancer patients undergoing surgery: A randomized controlled trial. Support. Care Cancer.

[32] Fincham G.W., Strauss C., Montero-Marin J., Cavanagh K. (2023). Effect of breathwork on stress and mental health: A meta-analysis of randomised-controlled trials. Sci. Rep..

[33] Islam H., Gibala M.J., Little J.P. (2022). Exercise Snacks: A Novel Strategy to Improve Cardiometabolic Health. Exerc. Sport Sci. Rev..

[34] Stamatakis E., Biswas R.K., Koemel N.A., Sabag A., Pulsford R., Atkin A.J., Stathi A., Cheng S., Thøgersen-Ntoumani C., Blodgett J.M. (2025). Dose Response of Incidental Physical Activity Against Cardiovascular Events and Mortality. Circulation.

