# Supplementary material for: Effects of Lifestyle-Integrated Active Pursed-Lip Diaphragmatic Breathing Training on the Prognosis of Chronic Heart Failure: A Retrospective Real-World Study
Source: Healthcare (Basel). 2026 Aug 11;14(16):2489. doi: 10.3390/healthcare14162489 (PMC13512455; doi:10.3390/healthcare14162489)
Supplement: Supplementary file 1 [file healthcare-14-02489-s001.zip › healthcare-4413829-supplementary.pdf]

Supplementary Table S1. Sensitivity analysis of LVEF: Comparison between complete-case analysis and multiple imputation analysis.

Variable / Statistic	Complete-case Analysis (Original Table 2)	Variable / Statistic (Sensitivity Analysis)
Respiratory Training Group		
Baseline LVEF, *n*	44.25 ± 17.40 (*n* = 16)	44.90 ± 17.11 (*n* = 20)
6-month LVEF, *n*	51.81 ± 14.89 (*n* = 16)	51.51 ± 14.48 (*n* = 20)
Within-group *P* value	0.003[a]	0.026[c]
Control Group		
Baseline LVEF, *n*	42.44 ± 14.23 (*n* = 27)	45.94 ± 14.88 (*n* = 36)
6-month LVEF, *n*	47.22 ± 13.34 (*n* = 27)	48.17 ± 12.88 (*n* = 36)
Within-group *P* value	0.022[a]	0.341[c]
Between-group Comparison (at 6 months)		
P value*	0.303[b]	0.402[d]

Abbreviations: LVEF, Left Ventricular Ejection Fraction. **Notes:** Data are presented as mean ± Standard Deviation (SD); **Complete-case analysis,** represents the results of paired *t*-tests utilizing only patients with complete paired data (i.e., those who had both baseline and 6-month follow-up LVEF records). Consequently, the sample sizes are n = 16 for the training group and *n* = 27 for the control group; **Multiple imputation analysis,** pooled estimates were derived using General Linear Models (GLM), with results combined across the 5 imputed datasets according to Rubin's rules. SD values in this column are approximated by averaging the SDs from the 5 imputed datasets. **Sample size discrepancy in Control Group,** the baseline sample size in the original study (Table 2) was 37. However, 1 patient in the control group died during the follow-up period and was excluded from the imputation model. Consequently, the multiple imputation analysis for the control group was based on 36 patients; [a]*P* values for within-group comparisons were derived from paired t-tests; [b]*P* values for between-group comparisons in the complete-case model were derived from independent t-tests; [c]*P* values for within-group comparisons in the imputed analysis were derived from GLM repeated-measures models pooled across datasets; [d]*P* values for between-group comparisons in the imputed analysis were derived from GLM independent-samples models pooled across datasets.

Supplementary Table S2. Between-Group Comparison of Core Secondary Outcomes at 6 Months Using Bootstrap ANCOVA.

Variable	Control (*n*)	Respiratory Training Group (*n*)	Adjusted Mean (SE)	Adjusted Mean Difference (95% BCa CI) *	Bootstrap *P*
6MWT distance, m	37	20	356.14 (17.96) vs 432.31 (24.57)	76.16 (7.64 to 141.17)	0.039
LVEF, %	27	16	47.22 (2.68) vs 51.81 (3.48)	4.59 (–4.28 to 13.16)	0.295
MLHFQ score	37	20	37.29 (2.88) vs 18.85 (3.93)	–18.44 (–27.71 to –9.79)	0.001

Abbreviations: ANCOVA, Analysis of Covariance; BCa, Bias-Corrected and Accelerated; CI, Confidence Interval; 6MWT, 6-minute walk test; LVEF, left ventricular ejection fraction; MLHFQ, Minnesota Living with Heart Failure Questionnaire; SE, standard error; CI, confidence interval. **Note**: *Adjusted mean differences and 95% BCa CIs were estimated using bias-corrected and accelerated bootstrap ANCOVA (1,000 resamples) with baseline values as covariates. Negative values for MLHFQ indicate greater improvement in quality of life (lower scores = better outcomes). Complete-case analysis was applied. Data are presented as adjusted mean (SE).

Supplementary Table S3. Univariate Cox Proportional Hazards Regression Analysis for MACE Across Baseline Characteristics.

Characteristics		Univariate Analysis	
		HR (95% CI)	*P* value
Smoking (Yes vs. No)		0.909 (0.336 – 2.458)	0.851
BMI (per 1 kg/m² increase)		0.953 (0.827 – 1.099)	0.510
Diabetes Mellitus (Yes vs. No)		0.976 (0.344 – 2.771)	0.964
Frailty Status (Yes vs. No)		1.726 (0.563 – 5.294)	0.340
6MWT distance (per 10-meter increase)		0.996 (0.963 – 1.031)	0.823
Handgrip strength (per 1-kg increase)		0.969 (0.909 – 1.034)	0.341
Serum Creatinine (per 1-μmol/L increase)		0.999 (0.994-1.005)	0.828
Medications	Diuretics (Yes vs. No)	0.950 (0.310 – 2.915)	0.929
	ARNI/ACEI/ARB (Yes vs. No)	0.771 (0.176 – 3.373)	0.730
	β-blocker (Yes vs. No)	0.826 (0.291 – 2.347)	0.720
	SGLT-2 inhibitor (Yes vs. No)	0.602 (0.173 – 2.096)	0.425

Abbreviations: 6MWT, 6-Minute Walk Test; ACEI, Angiotensin-Converting Enzyme Inhibitor; ARB, Angiotensin Receptor Blocker; ARNI, Angiotensin Receptor-Neprilysin Inhibitor; BMI, Body Mass Index; CI, Confidence Interval; EPV, Events Per Variable; HR, Hazard Ratio; MACE, Major Adverse Cardiovascular Events; SGLT-2, sodium-glucose cotransporter-2; **Note:** This table presents the complete univariate screening of baseline variables listed in Table 1. Variables with $P \geq 0.20$ or high collinearity with core prognostic markers were excluded from the main Table 5 to maintain model parsimony and adhere to the events-per-variable principle. NT-proBNP was excluded from all survival analyses due to a high missing rate and a non-random missingness mechanism. Continuous variables are expressed per unit increase as specified; categorical variables use the reference group indicated in parentheses.

Supplementary Table S4. Sensitivity Analyses for MACE-Free Survival Excluding Early Events.

Analytical Scenario	Respiratory Training Group (n / events) *	Control Group (n / events) *	Log-Rank *P*-value	Unadjusted Cox Regression HR (95% CI); *P*-value	Adjusted Cox Regression HR (95% CI); *P*-value [a]
Primary Analysis *(Full Cohort, $n = 58$)*	20 / 1	38 / 16	0.005	0.097 (0.013–0.729); *P* = 0.023	0.103 (0.014–0.779); *P* = 0.028
Sensitivity Analysis 1 *(Excluding Month-1 MACE)*	20 / 1	35 / 13	0.012	0.115 (0.015-0.878); $P = 0.037$	0.125 (0.016-0.963); $P = 0.046$
Sensitivity Analysis 2 *(Excluding Month-2 MACE)*	20 / 1	32/ 10	0.031	0.144 (0.018-1.126); $P = 0.065$	0.146 (0.019-1.143); $P = 0.067$

Abbreviations: MACE, Major Adverse Cardiovascular Events; HR, hazard ratio; CI, confidence interval; LVEF, left ventricular ejection fraction. **Note:** * Data are presented as the total number of patients at risk at the beginning of the scenario / the number of recorded events during the specified follow-up window; [a] Adjusted for baseline LVEF in the multivariable Cox proportional hazards model to adhere to the events-per-variable principle.

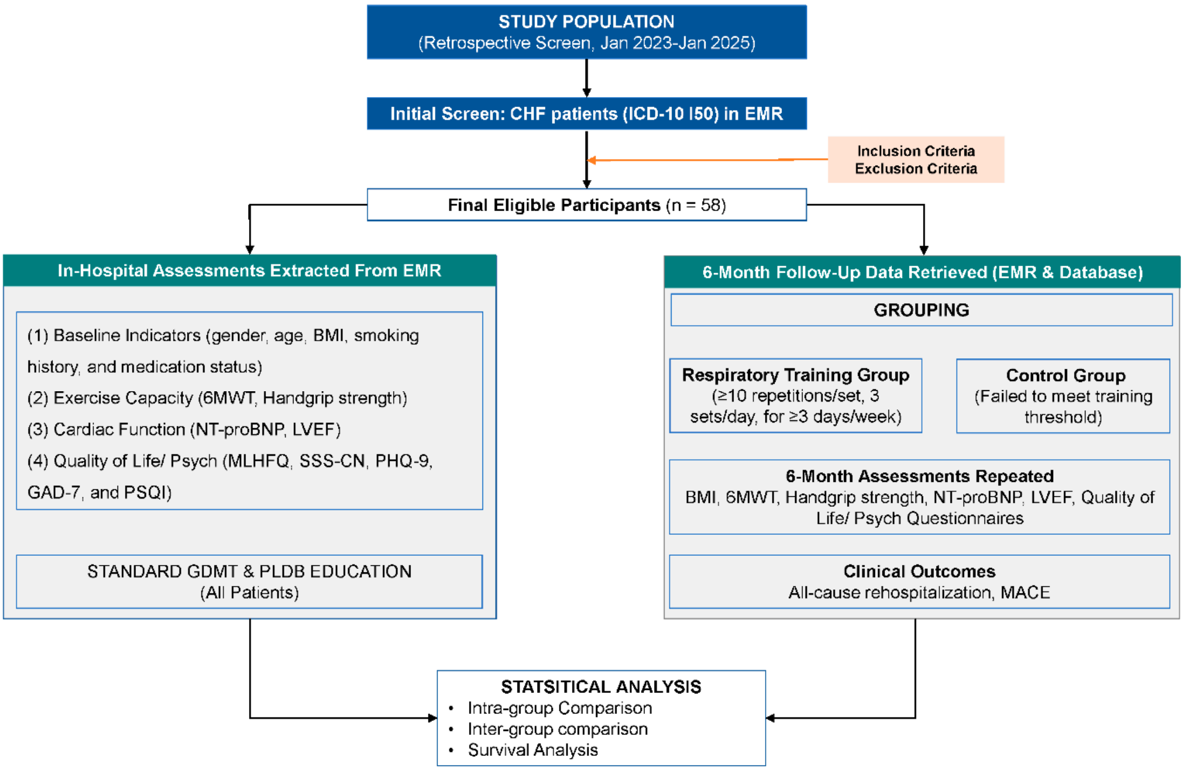

Supplementary Figure S1. Study design and flow chart.

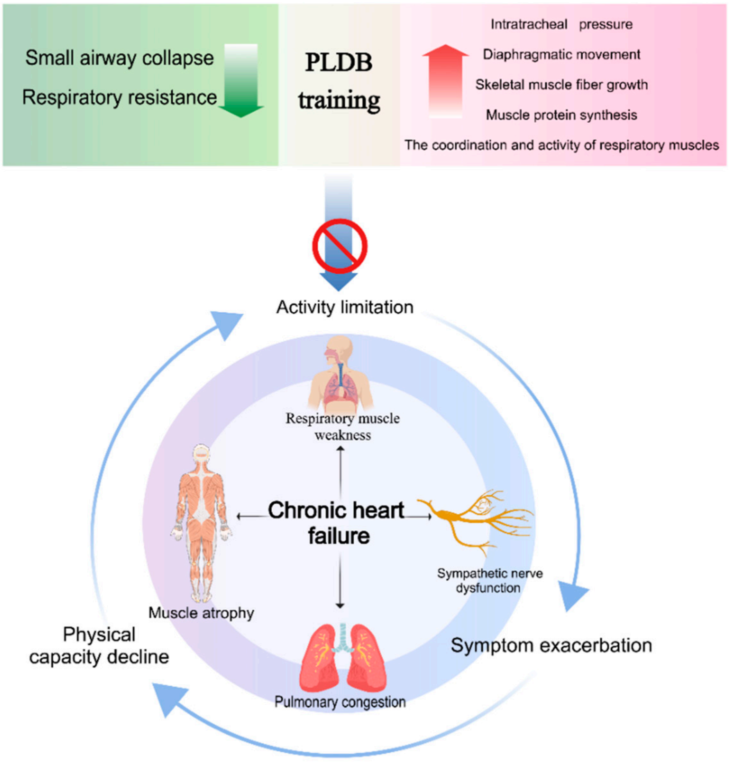

Supplementary Figure S2. Mechanism of PLDB Training in Blocking the "Activity Limitation-Physical Capacity Decline-Symptom Exacerbation" Vicious Cycle. Caption: The green box denotes the inhibitory effects induced by PLDB training, the red box signifies the promoting effects, and the concentric circle diagram located in the lower part of the figure elucidates the "activity limitation- physical capacity decline-symptom exacerbation" vicious cycle in chronic heart failure (CHF). Abbreviations: PLDB, Pursed-lip Diaphragmatic Breathing. The figure was created with MedPeer (medpeer.cn).
